# Impact of the first wave of the COVID-19 pandemic on cancer registration and cancer care: a European survey

**DOI:** 10.1093/eurpub/ckab214

**Published:** 2021-12-22

**Authors:** Luciana Neamţiu, Carmen Martos, Francesco Giusti, Raquel Negrão Carvalho, Giorgia Randi, Nadya Dimitrova, Manuela Flego, Tadeusz Dyba, Manola Bettio, Anna Gavin, Otto Visser, the ENCR Steering Committee, Anna Gavin, Otto Visser, María José Sánchez, Michael Eden, Fabrizio Stracci, Mario Šekerija, Maciej Trojanowski, Freddie Bray, Elizabeth van Eycken, Ana Miranda, Hans Storm

**Affiliations:** 1 European Commission, Joint Re search Centre (JRC), Ispra, Italy; 2 Northern Ireland Cancer Registry, Queens University, Belfast, UK; 3 Netherlands Comprehensive Cancer Organization (IKNL), Utrecht, The Netherlands

## Abstract

**Background:**

The coronavirus disease COVID-19 pandemic posed a number of challenges to the oncology community, particularly the diagnosis and care of cancer patients while ensuring safety from the virus for both patients and professionals: minimization of visits to the hospital, cancellation of the screening programmes and the difficulties in the management and operation of cancer registries (CRs) while working remotely. This article describes the effects in the medium term of the first wave of the COVID-19 pandemic on cancer registration in Europe, focusing on changes in cancer detection and treatment, possible reduction of CR resources and difficulties in the access to data sources.

**Methods:**

A questionnaire was distributed in June 2020 to the directors of 108 CRs from 34 countries affiliated to the European Network of Cancer Registries, providing a 37% response rate.

**Results:**

The results of the survey showed that cancer-screening programmes were mostly stopped or slowed down in the majority of regions covered by the respondent CRs. Cancer diagnostics and treatments were severely disrupted. The cancer registration process was also disrupted, due to changes in the work modalities for the personnel, as well as to the difficulties in accessing sources and/or receiving the notifications. In some CRs, staff was allocated to different activities related to controlling the pandemic. Several CRs reported that they were investigating the impact of COVID-19 on cancer care via dedicated studies.

**Conclusions:**

A careful analysis will be necessary for proper interpretation of temporal and geographical variations of the 2020 cancer burden indicators.

## Introduction

Since its onset, the COVID-19 pandemic has posed a number of challenges to the oncology community, particularly with regard to the management and operation of cancer-screening programmes, the diagnosis and care of cancer patients (in view of the need to minimize visits to the hospital or other healthcare facilities and the reduction in available intensive care beds for postoperative recovery due to their occupancy by COVID patients) and in ensuring the safety from the virus for both patients and professionals. As a consequence, at least during the first wave between March 2020 and May 2020, screening programmes were interrupted or slowed down,^1–3^ standard treatment modalities were disrupted^4^ and follow-up visits were postponed.^4^ The population-based cancer registries (CRs) are information systems designed for the collection, storage and management of data on persons with cancer. They collect information on all cancer cases that occurred in a defined population and provide statistics to support cancer control activities, enabling their overview in the registry area of operations. CRs are therefore the competent entities to allow quantification of the cancer care disruption impact at the population level. However, during the pandemic CR activities were also affected, following the increased call for resources in the response to the crisis, remote work and difficult access to data sources or notifications. In the European Union, at least four national population-based CRs reported a notable decrease in cancer-diagnosis notifications, namely the Netherlands,^2^ Slovenia,^3^ Denmark^5^ and Belgium.^6^ The Danish study determined a 20% reduction in cancer diagnosis, while the Belgian study reported a decrease of around 5000 cancer diagnoses in a 7-month period, roughly equivalent to the number of diagnoses occurring in 1 month under normal conditions. Also Northern Ireland publishes monthly data on impact of COVID-19 on cancer diagnosis on the Northern Ireland Cancer Registry website.^7^

## Aim

This article describes the effects in the medium-term of the first wave of the COVID-19 pandemic on cancer registration in Europe, focusing on cancer detection and treatment, possible reduction of CR resources and difficulties in the access to data sources.

## Methods

A questionnaire was distributed in June 2020 to the directors of 108 CRs affiliated to the European Network of Cancer Registries (ENCR) from 34 European countries. The survey was open until July 24. The survey was also promoted through the ENCR website. A reminder was sent 1 week before the deadline. The questionnaire was organized around four topics:

- general information about COVID-19 pandemic in the country or the region covered by the CR, and impact of the pandemic on cancer screening and treatment;
- predicted impact on medium-term cancer registration practices;
- participation of CRs in COVID-19-related studies; and
- collection of additional variables related to the assessment of the pandemic impact on cancer patients.

The detailed questionnaire is included only as Supplementary material.

## Results

**Figure 1:**
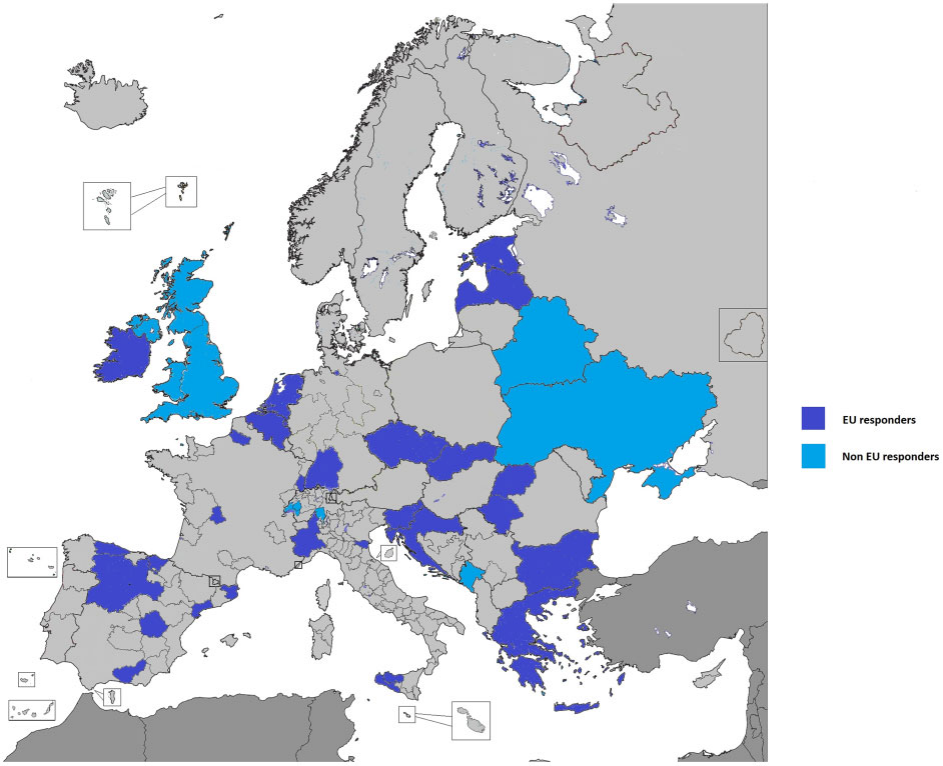
Geographical distribution of the Cancer registries responding to the JRC ENCR COVID-19 survey.

Forty CRs out of 108 (corresponding to a 37% response rate) from 22 countries in Europe, covering a population of 215 million inhabitants, responded to the questionnaire. These registries belong to 16 out of the 27 EU Member States (Belgium, Bulgaria, Croatia, Czechia, Estonia, France, Germany, Greece, Ireland, Italy, Latvia, the Netherlands, Romania, Slovakia, Slovenia and Spain), plus the UK, Switzerland, Belarus, Montenegro and Ukraine, as shown in figure 1. Registries replying to the questionnaire cover 35% of the EU population and 29% of the European population. All respondents with the exception of one are population-based registries. Four respondents consisted of childhood CRs (one of which is hospital based). The characteristic of the registries are reported in table 1.

**Table 1:** Number of respondent registries, population covered by the registries and corresponding population percentage by EU/non-EU belonging and national/regional registry area

Type of registry	Number of respondent registries	Population covered by the respondent registries	Percentage covered population (percentage of the population covered by the survey respondents out of corresponding population covered by registries affiliated to ENCR) (%)
EU National	10	66,156,714	54
EU Regional	17	32,813,083	28
Non-EU National	2	42,602,182	92
Non-EU Regional	7	68,275,179	89

*Note*: Childhood cancer registries are not considered in the table.

The results of the survey are presented here after according to the survey topics described in the Methods section.

### Lockdown and restrictive polices

Only four registries (Bulgaria, Latvia, the Netherlands and Belarus) reported that the registry area had not been under a formal lockdown (Note: By lockdown the authors mean the mobility of the population was restricted and people were more likely to stay at home) at the time of the survey. In contrast, in the majority of the cases, all other areas had been under lockdown, starting between 6 and 17 of March (reported by registries form Italy, France, Spain, Estonia, Slovenia, Romania, Ukraine, Switzerland, Germany, Belgium, the Netherlands, Czech Republic) or during the last week of March (Ireland, UK, Greece). The medical services resumed from end of April (Estonia) to June (restart of screening in Italy). Where lockdown was applied, and also in several countries without a formal lockdown, it was reported that non-urgent health care was postponed, and access to hospitals and outpatient care were severely disrupted.

### Cancer screening

Interruption or slowdown of organized population-based cancer screening (breast, cervical, colorectal) was reported in almost 90% of the areas covered by the respondent registries, according to the details in table 2. Replies from childhood CRs are not included in table 2 as they do not normally use screening programmes as source of information.

**Table 2:** Number of cancer registries (%) by status of the organized population-based cancer screening and cancer site

Screening program status	Breast cancer, *n* (%)	Cervical cancer, *n* (%)	Colorectal cancer, *n* (%)
Continued as usual	1 (3)	1 (4)	1 (4)
Interrupted	26 (84)	19 (76)	22 (82)
Slowed down	3 (10)	4 (16)	2 (7)
Information not provided	1 (3)	1 (4)	2 (7)
Total registries	31 (100)	25 (100)	27 (100)
No screening program in place	5	11	9

The disruption of cancer-screening programmes started at the beginning of March and the activities resumed between May and July. Cervical cancer screening was reported not to have resumed at all in Montenegro, due to the decision to reallocate the polymerase chain reaction devices [normally used for the human papilloma virus (HPV) screening] to the detection of  severe acute respiratory syndrome coronavirus 2 (SARS-CoV-2).

### Diagnostics and treatments

Non-urgent diagnostic visits for cancer were generally reported as disrupted from mid-March. The visits resumed in April for the areas covered by three registries, in May for the areas covered by nine CRs, and in June for the areas covered by the last three respondent registries. The remaining registries did not specify details regarding the resumptions.

Surgical treatment had been reported as disrupted by 51% of the registries, chemotherapy by 43%, immunotherapy by 44% and radiotherapy by 40%. The proportion of CRs without information on possible disruption for each treatment type was 21% for surgery, 13% for chemotherapy, 15% for immunotherapy and 20% for radiotherapy. In general, treatment was disrupted in the following countries: Belgium, Croatia, France, Ireland, Italy, Northern Ireland, Romania, Slovakia, Spain, Switzerland, Ukraine and UK. For bone marrow transplantation, many respondents (54%) could not report about the situation in their own area.

Recommendations from authorities were reported by 46% of respondents and consisted of general guidance on how to continue delivering health care under the on-going circumstances. In half of these situations, specific recommendations for oncology care had been issued.

### Disruptions in cancer registration processes

Among the registries having replied to the survey, 29% use only passive case reporting (cases reported by the information sources), 21% only active finding (CR staff collecting data from the information sources) and the remaining 50% adopt a mixed behaviour.

Among the 31 registries using a mixed or passive method, 58% reported no or not-significant disruption in receiving the notifications, 26% reported disruption when receiving notifications from selected sources and 16% reported disruption from all sources. The main sources of disruption were the pathology laboratories (100% of the six registries having reported) and the hospital discharge notifications (83%).

Among the 29 CRs using a mixed or active method, 56% of registries (from Belarus, Bulgaria, Czechia, Italy, Montenegro, The Netherlands, Slovakia, Slovenia, Switzerland, UK) did not report significant disruptions in accessing the sources.

The main reasons for disruptions of data collection were the remote setting for registries’ staff and related difficulties in remotely accessing the data sources. Moreover, disruption of services was reported in receiving the notifications, as well as lack of personnel due to re-allocation to other activities. However, the majority of the registries reported being able to recuperate fully or partially the missing data later on.

### Predicted impact on cancer burden indicators

Thirty-eight percent of the CRs reported that they did not expect a decrease in 2020 cancer-incidence rates. On the contrary, 21% expected a drop for all cancers, 15% for selected cancer entities (due to disruptions in the screening programmes), while 26% did not have expectations.

More than half of the CRs (56%) reported evidence of impact on data processing (abstracting, checking, validating, coding data, etc.), mainly due to the reduction in personnel and to the limitations in accessing resources or databases when working remotely.

Selected CRs (18% from Belgium, the Netherlands, Slovenia, Switzerland, Spain, UK) were planning to process 2020 data (partially or totally) earlier than usual, to estimate the impact of the pandemic in cancer indicators.

As for expected impact in the survival, changes in cancer-survival ratio for cases diagnosed in 2020 were estimated for all cancers by 10% of the CRs, while 21% of respondents predicted impact only in selected cancer entities (mainly for cancers with screening programmes in place). More than half of the registries could not predict about the possible impact on survival and 18% did not expect changes.

### Involvement of CRs in research related to COVID-19

Among the respondents, 43% of the CRs (from Belarus, Belgium, Croatia, Estonia, Germany, Ireland, Italy, the Netherlands, Slovenia, Spain, Switzerland, UK) at the time of the survey were already carrying out research or were involved in studies to assess the impact of COVID-19 in cancer diagnosis and care. Reported investigation topics were as follows: changes in incidence, stage and treatment, delay in diagnosis and treatment, impact on cancer care and patients' social support, impact on mortality for other causes due to disrupted access to the usual health care, serological prevalence of COVID-19 in cancer patients, impact on outcome of cancer screening programmes, measurement of excess mortality in prevalent cancer cases, assessment of patterns in incidence trends by cancer type.

On top of this, 18% of the registries at the time of the survey were collecting additional variables on top of the standard, to support the quantification of the COVID-19 impact of in cancer registration and care (e.g. delays in diagnosis and treatment, modification of treatment, etc.). Lastly, 13% of the registries were collecting additional variables related to diagnosis and treatment, focused specifically on patients with SARS-CoV-2 infection as comorbidity.

CRs reported their willingness to perform or collaborate on projects evaluating the impact of the pandemic in cancer screening, diagnosis and care, as well as in cancer registration, under the condition of being supported financially for the work.

## Discussion

The article gives an overview of the impact of the first wave of COVID-19 pandemic in cancer screening, diagnosis and care, as well as on CRs operations in Europe.

The results of the survey show that cancer-screening programmes were mostly stopped or slowed down in the majority of regions covered by the respondent CRs, for at least 2 months. Moreover, within 4 months from the beginning of the pandemic, some registries had already taken the initiative to embark on studies for assessing its consequences.

Cancer diagnosis and treatment were severely disrupted; among treatment regimes, surgery was the most affected.

To mitigate the effect of the pandemic on cancer diagnosis and care, medical oncology societies, e.g. European Society for Medical Oncology,^4^ and European Society for Paediatric Oncology^8^ issued recommendations on how to prioritize diagnosis and treatment. In this context, CRs data could contribute to evaluate the impact of the diagnosis and treatment modification at the population level due to the pandemic and to analyze possible modifications in cancer indicators and outcomes related to changes in the diagnosis and treatment.

At the time of the survey, a number of registries were already participating in or conducting studies to measure the impact of the COVID-19 in cancer care. Preliminary results showed a relative decrease in cancer diagnosis by 25% (from January to end of March 2020) for all cancers, and 39% for skin cancers in the Netherlands.^2^ Similar figures were reported in Slovenia, with ‘a third fewer cases diagnosed in April compared with an average pre-epidemic period’.^3^ In Denmark, for all-cancers incidence ‘the combined reduction from March to May was 33% [95% confidence interval (CI): 26–40)’.^5^ In Northern Ireland, ‘during first 6 months of the COVID-19 pandemic, pathologic diagnoses of Barrett’s oesophagus fell by 59.3% compared with historical rates, with a 95.5% decline in April alone. The suspension of endoscopy services, disruption to clinical activity and decline in presentation of symptomatic patients also led to a 26.6% fall in esophagogastric cancer diagnoses’.^9^

Also a study from Northern Portugal completed with CR data linked with clinical files showed that ‘significantly higher adjusted hazards of death were observed for patients with stage III cancer [hazard ration (HR) = 2.37; 95% CI 1.14–4.94] and those undergoing surgical treatment (HR = 3.97; 95% CI 1.14–13.77) or receiving radiotherapy (HR = 1.96; 95% CI 1.96–3.74), while patients who did not receive any treatment had lower mortality hazards (HR = 0.62; 95% CI 0.46–0.83)’.^10^

Observational studies performed using other sources than CRs showed also a decrease in cancer treatment: in a cancer centre in Poland,^11^ the number of patients undergoing daily radiotherapy treatment was 37% less than prior to the pandemic. Moreover, in the whole of Poland, the number of patients directed to the fast oncological diagnosis and treatment path, measured by the number of issued oncology diagnoses and treatment cards (ODaTCs), decreased. For instance, considering solely breast cancer in the period between January and April 2020, the number of issued ODaTCs decreased by 45%.^12^ In England, the National Radiotherapy Dataset concluded that ‘by diagnosis, the largest reduction from 2019 to 2020 in treatment courses was for prostate cancer (77.0% in April) and non-melanoma skin cancer (72.4% in April)’.^13^ In Northern Ireland, ‘data from the four Northern Ireland pathology labs showed a 23% reduction in cancer diagnoses compared to the same time period in the preceding three years’ ‘between 1 March and 12 September 2020’.^14^ Overall in Northern Ireland, a 41% overall reduction occurred in pathologically diagnosed cancers by June 2020, compared with similar period in previous years.^7^ A study conducted by the Spanish Association against Cancer and several cancer scientific societies during the first pandemic wave, based on a survey of 37 tertiary centres across the whole country, showed less significant reductions such as a 9.5% decrease in chemotherapy treatments and a 5% decrease in radiotherapy treatments. The same study showed a 21% reduction in cancer diagnoses and significant fall in cytologies (57%) and biopsies (41%). These results are based on the comparison between the period March and June 2020 and the same time window in 2019.^15^

Around 20% of the survey, respondents reported the intention to process 2020 data faster than usual, to allow analysis on impact of the pandemic in cancer diagnosis.

The information collected from CRs shows that diagnostic visits and specific treatments were mostly affected, as a consequence of the pandemic. This is predicted to lead to a lower number of cancer incident cases and to increases in the stage at diagnosis, as well as negatively impacting on survival. A careful analysis will be necessary for proper interpretation of temporal and geographical variations of the 2020 cancer burden indicators.^16^ The disruption in the vaccination programs for children (e.g. hepatitis B and HPV)^17^ should be also taken into account in future predictions.

Similar results at worldwide level are reported by the International Agency for Research in Cancer (IARC)^18^ supporting the need to continue and enhance the cancer surveillance.

Concerning the non-communicable diseases (NCD), the results of a World Health Organization (WHO) survey conducted in May 2020^19^ showed ‘the considerable impact of the COVID-19 pandemic on services for NCD prevention, treatment and rehabilitation in countries in the WHO European Region. Overall, countries in the Region have patterns similar to those at the global level in disruption of services’.

The article has some limitations. Some areas in Europe, such as Northern Europe, were not represented in the survey, due to lack of participation possibly because the timing of the survey that coincided with the summer holidays in those countries. Respondents to the survey cover almost one third of the European population. The coverage was still below 50% even after a reminder was sent. The low participation rate might be due to the remote work and allocation of registries’ staff to COVID-19 crisis. However, the results gathered through the survey are confirmed by studies published by the registries.^2^^,^^3^^,^^6^^,^^7^^,^^9^ Despite the limited coverage, the study reports the first results at the European level comparing several countries with different situations both in terms of registries’ organization and COVID-19 burden. To complement and follow-up, a second survey is ongoing.

## Supplementary data

Supplementary data are available at *EURPUB* online.

## Supplementary Material

ckab214_Supplementary_Data

## Members of the ENCR Steering Committee 2018–2020

Anna Gavin^1^, Otto Visser^2^, María José Sánchez^3^, Michael Eden^4^, Fabrizio Stracci^5^, Mario Šekerija^6^, Maciej Trojanowski^7^, Freddie Bray^8^, Elizabeth van Eycken^9^, Ana Miranda^10^, Hans Storm.^11^

^1^Northern Ireland Cancer Registry, Queens University, Belfast, UK; ^2^Netherlands Comprehensive Cancer Organization (IKNL), Utrecht, The Netherlands; ^3^Granada Cancer Registry, Andalusian School of Public Health; Instituto de Investigación Biosanitaria (ibs.GRANADA); Centro de Investigación Biomédica en Red de Epidemiología y Salud Pública (CIBERESP); ^4^National Cancer Registration and Analysis Service, London, UK; ^5^Umbria Cancer Registry, Perugia, Italy; ^6^Croatian National Cancer Registry, Croatian Institute of Public Health, Zagreb, Croatia; ^7^Greater Poland Cancer Centre, Greater Poland Cancer Registry, Poznan, Poland; ^8^International Agency for Research on Cancer (IARC), Lyon, France; ^9^Belgian Cancer Registry, Brussels, Belgium; ^10^Portuguese Institute of Oncology Francisco Gentil, Lisbon, Portugal; ^11^Danish Cancer Society, Copenhagen, Denmark.
