# Supplementary material for: Impact of the first wave of the COVID-19 pandemic on cancer registration and cancer care: a European survey
Source: Eur J Public Health. 2021 Dec 22;32(2):311–5. doi: 10.1093/eurpub/ckab214 (PMC8975538; doi:10.1093/eurpub/ckab214)
Supplement: ckab214_Supplementary_Data [file ckab214_supplementary_data.pdf]

# JRC ENCR Questionnaire on the impact of the COVID-19 pandemic on cancer registration

Fields marked with * are mandatory.

**Please choose the language from the drop list on the right side of the questionnaire**

## JRC ENCR Questionnaire on the impact of the COVID-19 pandemic on cancer registration

**Background**
With the rapid evolution of the COVID-19 pandemic, the oncology world is facing some challenges: screening program management, how to diagnose and provide best available care while minimising the visits in the hospital (or other healthcare facilities) and maximising the safety for both patients and professionals. Screening programs are halted, treatment modalities are modified and follow-up visits are postponed.

**Aim**
The survey's goal is to assess the impact of COVID-19 pandemic on cancer registration medium and long term activities and connected with the impact on cancer detection and treatment.

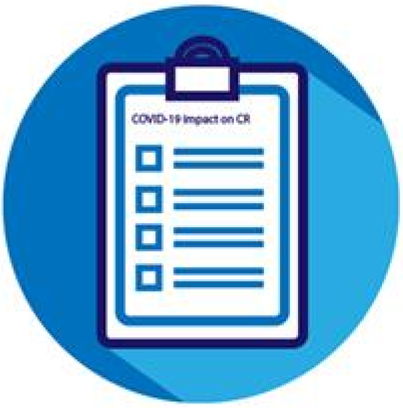


## 1. Information about the registry

* 1.1. Name of the registry

* 1.2. Country

* 1.3. Geographical area (e.g. country, region, province etc) covered by the registry

## 2. Contact details of the person completing this questionnaire

* 2.1. First Name

* 2.2. Last Name

* 2.3. Contact e-mail

## 3. General information about COVID-19 pandemic in the country or the region covered by the cancer registry and the impact on cancer screening, diagnosis and treatment

* 3.1. Was the area covered by the registry under lockdown?

- Yes
- No

* If yes, starting date

* Please describe the phases of the ending of lockdown in relation with the impact for cancer care (e.g. starting access to hospitals, outpatient care, rehabilitation facilities etc.)

* 3.2.1. In the area covered by the registry organised screening program for breast cancer

- continued as usual
- was interrupted
- was slowed down
- I don't know
- not applicable

* Please provide the period when the breast cancer screening program was interrupted

* 3.2.2. In the area covered by the registry organised screening program for cervical cancer

- continued as usual
- was interrupted
- was slowed down
- I don't know
- not applicable

* Please provide the period when the cervical cancer screening program was interrupted

* 3.2.3. In the area covered by the registry organised screening program for colorectal cancer

- continued as usual
- was interrupted
- was slowed down
- I don't know
- not applicable

* Please provide the period when the colorectal cancer screening program was interrupted

* 3.3. In the area covered by the cancer registry, diagnostic visits for cancer

- continued as usual
- were disrupted
- I don't know

* Please detail the period when the diagnostic visits were disrupted, as well as a short description of the disruption (no availability for non COVID-19 patients, percentage of centers affected etc.)

* 3.4. In the area covered by the cancer registry, surgical treatment for cancer patients

- continued as usual
- was disrupted (canceled, delayed, etc)
- I don't know

* Please detail the period(s) when the surgery treatment was disrupted

* 3.5. In the area covered by the cancer registry, chemotherapy treatment for cancer patients

- continued as usual
- was disrupted (canceled, delayed, modified etc)
- I don't know

* Please detail the period(s) when the chemothrapy treatment was disrupted

* 3.6. In the area covered by the cancer registry, immunotherapy treatment for cancer patients

- continued as usual
- was disrupted (canceled, delayed, modified etc)
- I don't know

* Please detail the period(s) when the immumotherapy treatment was disrupted

* 3.7. In the area covered by the cancer registry, bone marrow transplant for cancer patients

- continued as usual
- was disrupted (canceled, delayed, etc)
- I don't know

* Please detail the period(s) when the bone marrow transplant for cancer patients was disrupted

* 3.8. Were there any national/regional recommendations from the authorities about cancer diagnostic /treatment strategies during the lockdown or since start of the COVID-19 crisis?

- Yes
- No
- I don't know

* Please detail

* 3.9. In the area covered by the cancer registry, radiotherapy treatment for cancer patients

- continued as usual
- was disrupted (canceled, delayed, modified etc)
- I don't know

* Please detail the period(s) when the radiotherapy treatment was disrupted

# 4. Direct impact on short and medium term cancer registration

## 4.1.Data collection

* 4.1.1. In your registry, data collection is
- passive
- active
- mixt

* Since COVID-19 pandemic started, the registry had disruption in receiving the notifications
- Yes, from all sources
- Yes, from some sources
- No or not significant disruption

* Please detail the sources with disruption in notifications (e.g. pathology labs, death certificates, hospital discharge, etc.)

* Since COVID-19 pandemic started, the registry had disruptions in accessing the sources
- Yes, all sources
- Yes, some sources
- No or not significant disruption

* Please detail the sources with disruption in access (e.g. pathology labs, radiotherapy departments, etc.)

* Reason for disruptions in data collection
- The registry personnel was allocated to other activities (e.g. to support the response to COVID-19 crisis)
- The registry personnel was working from home
- The providers of the notifications were not sending the notifications
- Access to sources was not possible / restricted
- Other

* Please detail

* If the collection of data was disturbed, can the registry recuperate the information later?
- Yes
- No
- Partially

* 4.1.2. Do you expect to see a decrease in cancer incidence rates in your registry area?
- Yes, for all cancers
- Yes, for some cancers
- No
- I don't know

* Please detail cancer (s) for which you expect a decrease

* If yes, possible reasons for decrease in your country/ region
- Decrease in reporting of cases
- Decrease in access to sources
- Disruptions in screening programs
- Disruptions in diagnostic visits
- Decrease of referrals for diagnosis
- Less people at risk (e.g. high number of excess death due to COVID-19)
- Other

* Please enumerate other reasons

* 4.1.3. Do you have access to death certificates?
- Yes
- No

* Are you collecting cause of death?
- Yes
- No

* Is your registry registering
- leading cause of death
- multiple cause of death

* If not, can you collect for specific studies all causes of death?
- Yes
- No

* Please detail

* Do you expect to see changes in cancer survival ratio for cases diagnosed in 2020 in your registry area?
- Yes, for all cancers

- Yes, for some cancers
- No
- I don't know

* Possible reasons
- [ ] Modification in treatment
- [ ] Less access to treatments
- [ ] Other

### 4.2.Data processing

* 4.2.1. Has the COVID-19 pandemic had any impact on data processing (abstracting, checking, validating , coding data etc.)?
- Yes
- No or not significant
- I don't know

* Reasons for impact in data processing
- [ ] Less personnel to process data
- [ ] Unavailability of some data sources in due time
- [ ] Other

* Please detail

* 4.2.2. Is your registry planning to process 2020 data (or a part of 2020 data) earlier (fast track process)?
- Yes
- No
- I don't know

* If yes, when you estimate to have it processed?

* 4.2.3. Is your cancer registry performing research / is involved in studies on impact of COVID-19 in cancer diagnosis and care?
- Yes
- No
- I don't know

* If yes, please detail the study (ies) the registry is involved.

* 4.2.4. Is your registry collecting specific information regarding cancer patients infected with SARS-CoV-2?

- Yes
- No

* Please detail the variable(s) collected

* 4.2.5.Is your registry collecting additional variables to measure the impact of COVID-19 in cancer registration and care (e.g. delays in diagnosis and treatment, modification of treatment etc.)

- Yes
- No

* Please detail the variable(s) collected

Do you have any additional details/ comments regarding the impact of the COVID-19 pandemic on the cancer registration and cancer diagnosis and care?

*2000 character(s) maximum*

- [ ] I accept the privacy statement

Show
